# Editorial: New Horizons in Cellular Optogenetics

**DOI:** 10.3389/fncel.2022.875602

**Published:** 2022-03-23

**Authors:** Suneel Kateriya, Sushil K. Jha, Andreas Möglich

**Affiliations:** ^1^School of Biotechnology, Jawaharlal Nehru University, New Delhi, India; ^2^School of Life Sciences, Jawaharlal Nehru University, New Delhi, India; ^3^Photobiochemie, Universität Bayreuth, Bayreuth, Germany

**Keywords:** channelrhodopsin, neurons, neurosciences, optogenetics, sensory photoreceptor, signal transduction

Optogenetics is a powerful research tool that, over the past two decades, has dramatically advanced our understanding of various forms of neuronal signaling including identification of brain circuits underlying different behaviors. At the heart of these advancements lie sensory photoreceptors that serve as genetically encoded actuators to trigger cellular and organismal responses by light. Beyond the naturally occurring variety, artificial photoreceptors have made additional biological processes amenable to optogenetic intervention. An ever-increasing armory of optogenetic tools now stands ready to decipher complex neural networks, behavior, signaling cascades, genome editing, and many more cellular events at the molecular level. Beyond actuators, the modern optogenetic toolkit also encompasses genetically encodable optical sensors for real-time monitoring of cellular events. This Research Topic collects contributions from leaders in the field who have devised and applied optogenetic tools to unravel different aspects of neural signaling.

The seminal discovery of channelrhodopsins (ChR) as light-gated ion channels in flagellate algae ushered in optogenetics and enabled its revolutionary applications in the neurosciences. In the last two decades, cohorts of functionally diverse ChRs have been discovered in nature or devised by protein engineering, thus greatly adding to the optogenetic arsenal. In their article, Govorunova et al. survey the rich repertoire of currently identified ChRs, their phylogeny, and taxonomic distribution. As a group, ChRs exhibit remarkable diversity in ion selectivity, conductance, photodynamic characteristics, and color-tuning. Atomically resolved ChR structures, becoming ever more readily available, benefit the molecular rationalization of these traits. Structure-function relationships thus established pave the way toward the (semi-) rational engineering and optimization of customized ChRs. Tailor-made ChRs with desired properties stand to further empower optogenetics and may prove decisive for translational applications in clinical settings (e.g., for the treatment of retinitis pigmentosa, epilepsy, or cardiac arrhythmia).

ChRs also take center stage in the contribution by Schilardi and Kleinlogel, who explore cell-specific optogenetic regulation of the physiology of bipolar cells (BC). Pertinent approaches are currently harnessed in optogenetic gene therapy for a specific type of blindness. By deciphering mechanistic details of BC physiology using optogenetic modalities, a novel therapy for the cure of blindness and retinal degeneration can potentially be developed.

Real-time observation of neural activity and the stimulation thereof with cellular resolution greatly ease the analyses of sensory processing and associated physiological functions. In this regard, Narcisse et al. utilize a combination of an optogenetic modulator and a bioluminescent Ca^2+^ sensor for continuous monitoring of neural activities in the visual cortex, with high spatio-temporal resolution. The application of artificial intelligence identifies parameters for neural activation, which in turn transform Ca^2+^ bioluminescence signals into neural network activity signatures. The combination of bioluminescence, multi-characteristic opsins, and artificial intelligence stands to be useful for regulating and monitoring large-scale activities in the brain.

Piqued by the far-reaching and rapid impact optogenetics had upon its inception, researchers also evaluated sensory photoreceptors other than opsins for their application potential. As reviewed by Lehtinen et al., optogenetic actuators that respond to red light are of particular interest in this regard as long wavelengths exhibit superior penetration of biological tissues compared to short wavelengths. Beyond certain rhodopsins responsive to red light, it is foremost the phytochrome superfamily which offers sensitivity to red and far-red (i.e., near-infrared) light. The authors discuss diverse optogenetic approaches by which cellular state and physiology can be manipulated by red and far-red light. Moreover, practical aspects relevant to application, such as gene, chromophore, and light delivery, are treated. In closing, a critique of the current state of red-light optogenetics provides pointers for the future.

Along related lines, Zhu et al. review the application of non-opsin photoreceptors to the study of cells, neurons, and disease. By complementing the established rhodopsin ion channels and pumps, these photoreceptors unlock further cellular processes for optogenetics beyond the well-established light-regulated manipulation of membrane potential. Various optogenetic implements can thus be leveraged for modulating genome editing and regulation, cell and organ development and differentiation, cellular function, and synaptic communication. The underlying optogenetic strategies principally translate to the investigation of neurological disorders and, prospectively, to their treatment. The authors discuss recent progress to this end, for instance in cellular therapies, and identify challenges that need to be met for therapeutic applications, including gene and light delivery (also see Lehtinen et al.).

As also noted by Zhu et al., one of the most versatile means to subject target protein activity to light control is provided by light-activated, dynamic protein-protein interactions. As a case in point, Chen et al. apply a light-oxygen-voltage (LOV) photoreceptor pair that dimerizes under blue light to reconstitute the activity of a split biotin ligase. Spatial control and temporal acuity are thus installed in the enzyme, nonspecific background activity is markedly reduced in cell culture, and the analysis of intracellular protein-protein interactions is facilitated. To sensitively monitor intracellular biotinylation, Chen et al. also furnish a genetically encoded sensor by adapting and optimizing a monomeric streptavidin variant linked to a fluorescent reporter protein. Taken together, the authors' work will benefit the study of functionally relevant protein-protein interactions by proximity proteomics.

Overall, the articles within this Research Topic showcase existing applications and the potential of optogenetics in the neurosciences. In the future, a better understanding of cellular trafficking, localization, and site-specific functions of optogenetic actuators and sensors, in particular of the transmembrane rhodopsins, is required. To this end, native and engineered cellular signal sequences need to be delineated and evaluated. Signal sequences for subcellular targeting of optogenetic payloads to the nucleus, mitochondria, lysosome, cilia, axon, dendrites, and synapses are of particular interest, as they will facilitate the analyses of ciliopathies, channelopathies, and neural diseases.

## Author Contributions

SK, SJ, and AM conceived of the Research Topic, edited submitted manuscripts, and wrote the article. All authors contributed to the article and approved the submitted version.

## Conflict of Interest

The authors declare that the research was conducted in the absence of any commercial or financial relationships that could be construed as a potential conflict of interest.

## Publisher's Note

All claims expressed in this article are solely those of the authors and do not necessarily represent those of their affiliated organizations, or those of the publisher, the editors and the reviewers. Any product that may be evaluated in this article, or claim that may be made by its manufacturer, is not guaranteed or endorsed by the publisher.

